# Secure Messaging Use and Wrong-Patient Ordering Errors Among Inpatient Clinicians

**DOI:** 10.1001/jamanetworkopen.2024.47797

**Published:** 2024-12-04

**Authors:** Sunny S. Lou, Daphne Lew, Linlin Xia, Laura Baratta, Elise Eiden, Thomas Kannampallil

**Affiliations:** 1 Department of Anesthesiology, Washington University School of Medicine in St Louis, St Louis, Missouri; 2 Institute for Informatics, Data Science and Biostatistics, Washington University School of Medicine in St Louis, St Louis, Missouri; 3 Division of Computational and Data Sciences, Washington University in St Louis, St Louis, Missouri; 4 Roy and Diana Vagelos Division of Biology and Biomedical Sciences, Washington University School of Medicine in St Louis, St Louis, Missouri; 5 Department of Computer Science and Engineering, Washington University in St Louis, St Louis, Missouri

## Abstract

**Importance:**

Use of secure messaging for clinician-to-clinician communication has increased exponentially over the past decade, but its association with clinician work is poorly understood.

**Objective:**

To investigate the association between secure messaging use and wrong-patient ordering errors.

**Design, Setting, and Participants:**

This cohort study included inpatient attending physicians, trainee physicians, and advanced practice practitioners (APPs) from 14 academic and community hospitals. Secure messaging volume was assessed over a 3-month period (February 1 to April 30, 2023).

**Exposure:**

Secure messaging volume per clinician-day, measured as the count of secure messages sent and received by a clinician on a given clinician-day.

**Main Outcomes and Measures:**

Retract-and-reorder events were used to identify wrong-patient ordering errors, and the presence of any retract-and-reorder event on a clinician-day was the primary outcome. Multilevel logistic regression was used to examine the association between secure messaging volume and wrong-patient ordering errors after adjusting for clinician age, sex, patient load, order volume, and clinical service.

**Results:**

A total of 3239 clinicians (median [IQR] age, 37 [32-46] years; 1791 female [55.3%]; 1680 attending physicians [51.2%], 560 trainee physicians [17.3%], and 999 APPs [30.8%]) with 75 546 clinician-days were included. Median secure messaging volume was 16 (IQR, 0-61) messages per day. Retract-and-reorder events were identified on 295 clinician-days (0.4%). Clinicians with secure messaging volume at the 75th percentile had a 10% higher odds of wrong-patient ordering errors compared with those at the 25th percentile (odds ratio [OR], 1.10; 95% CI, 1.01-1.20). After stratifying by clinician role, the association between secure messaging and wrong-patient ordering errors was observed only for attending physicians (OR, 1.20; 95% CI, 1.02-1.42) and APPs (OR, 1.18; 95% CI, 1.00-1.40).

**Conclusions and Relevance:**

In this cohort study of inpatient clinicians, higher daily secure messaging was associated with increased odds of wrong-patient ordering errors. Although messaging may increase cognitive load and risk for wrong-patient ordering errors, these results do not provide conclusive evidence regarding the direct impact of secure messaging on errors, as increased messaging may also reflect greater care coordination, increased patient complexity, or communication of the presence of a wrong-patient ordering error.

## Key Points

**Question:**

What is the association between secure messaging volume and wrong-patient ordering errors?

**Findings:**

In this cohort study of 3239 inpatient clinicians and 75 546 clinician workdays, increased daily secure messaging volume was associated with increased odds of wrong-patient ordering errors. After stratifying by clinician role, the odds were highest for attending physicians followed by advanced practice practitioners.

**Meaning:**

These findings suggest that secure messaging use may have implications for patient safety.

## Teaser

This cohort study uses electronic health record data to investigate the association between hospital clinician use of secure messaging and wrong-patient ordering errors.

## Introduction

Communication is a crucial part of health care delivery, and failures in communication have been identified as among the leading causes of sentinel events in the US.^1^ Much of the prior research on health care communication has focused on face-to-face communication, highlighting the risks associated with omission of information or the transfer of erroneous information.^2^ For example, miscommunication has been implicated as a leading contributor to many patient safety events.^3^

Historically, much of health care communication has relied on synchronous communication (eg, face-to-face, telephone); however, the advent and adoption of mobile technologies and changes in federal regulations regarding clinician messaging^4^ have rapidly led to the use of asynchronous text-based communication.^5,6,7^ Over the past 5 years, there has been an exponential increase in the use of secure messaging, with some reports suggesting that the volume of messages exceeds 1.5 million per month.^8,9^

Considerable concerns exist regarding the influence of this new form of communication on health care delivery. For example, such messaging applications have been shown to correlate with clinician workload, cognitive burden, and interruptions.^10^ In a recent study, our group found that increasing secure messaging volume contributed to increased time spent using the electronic health record (EHR) per day and to increased attention switching (a proxy for cognitive burden) (D. Lew, PhD, written communication; July 1, 2024). Other investigators have highlighted the potentially interruptive nature of secure messaging, with studies showing that the median response time for a message is approximately 2 minutes.^9^

Although there is widespread use and adoption of secure messaging, studies investigating the implications of secure messaging for patient-related or safety outcomes are largely absent in the literature. In this study, we investigated the association between secure messaging use and wrong-patient ordering errors, an important patient safety outcome, among inpatient attending physicians, trainee physicians (residents and fellows), and advance practice practitioners (APPs).

## Methods

### Setting and Participants

This cohort study included data from 14 hospitals associated with the Washington University School of Medicine and the BJC HealthCare system, serving a diverse population in Missouri and Illinois. These hospitals included both academic and community practice settings and used the Epic EHR (Epic Systems). Epic’s integrated secure messaging platform, Secure Chat, has been available for use across the health system since 2019 and allows users to securely send text-based messages to other Epic EHR users within the system. Both the desktop and mobile versions of Secure Chat are available to clinicians; messages on both interfaces appear in a threaded fashion and allow clinicians to optionally attach a patient identifier (eg, a medical record number) for direct access to the patient record. In the desktop version, the messages appear as a pop-up window, whereas users of the mobile version receive a push notification. This study was approved by the institutional review board of Washington University with a waiver of informed consent as the research was considered minimal risk. We followed the Strengthening the Reporting of Observational Studies in Epidemiology (STROBE) reporting guideline.

For this study, we included all attending physicians, trainee physicians (residents, fellows), and APPs (nurse practitioners and physician assistants) who worked in an inpatient setting at 1 of the 14 hospitals during the 3-month study period from February 1 through April 30, 2023. Ten clinicians with missing demographic information were removed from the analysis.

### Data Sources

There were 3 data sources used for this study: secure messaging metadata, retract-and-reorder (RAR) events, and EHR-based audit logs. Data were collected on the use of Epic’s Secure Chat for the study period from Epic’s Clarity database. For each message, data included the message sender, message recipient, time at which the message was sent, time at which the message was responded to (if a response existed), the conversation thread the message was associated with, whether a patient medical record number was attached to the message (a binary indicator), and the message character length. Message content was not retrieved for this study. Additional information on clinicians’ role (eg, attending, resident, APP) and their clinical service location (ie, clinical setting in which they worked on a daily basis [eg, the intensive care unit]) was also retrieved.

We captured the RAR events over the study period using a custom Epic Clarity query. An RAR event is triggered when an order is canceled by an ordering clinician within the first 10 minutes of the order and it is reordered by the same clinician within the next 10 minutes for a different patient.^11,12^ An RAR event is often used as a surrogate measure for wrong-patient ordering errors and has been endorsed as a validated measure by the National Quality Forum and the Office of the National Coordinator for Health Information Technology.^13,14^ The RAR measure has been extensively used to study wrong-patient ordering errors in a variety of settings.^15,16^

We retrieved EHR-based audit logs for the study period from the ACCESS_LOG table in Epic’s Clarity database. EHR-based audit logs (ie, trails of clinician activities in the EHR) are mandated for compliance with the Health Information Portability and Accountability Act. These data have been extensively used to develop clinician-centered perspectives of clinical work activities, workflows, and clinician behaviors.^17,18,19^

### Data Preprocessing

Secure messaging data, RAR events, and audit logs were organized and aggregated at the clinician-day level. A clinician-day was defined as a 24-hour period for each calendar date. The criteria for including a specific clinician-day were the following: if the clinician signed at least 1 patient note, and put in at least 1 order, and had at least 500 audit log actions during a day. Prior studies have shown that on days that clinicians work, they log more than 500 audit log actions.^19^ The measures for notes signed, orders placed, and number of audit log actions were computed directly from EHR-based audit logs for the study period.

As it was possible that a clinician worked in multiple settings over the study period, we also recorded the clinical service for each clinician-day. For example, it is possible that a trainee physician worked at a hospital medicine service for 1 week and at an intensive care unit the following week. We used the clinician login information in the Epic EHR to ascertain the clinical service. When logging into the EHR, clinicians have to select a context for their login that provides a customized service-specific interface aligned with the clinical service. We used the most frequent login context for each clinician-day as a proxy for their clinical service assignment for that day.

Patient load was measured as the total number of unique patients for whom a clinician signed a note per day, as clinicians are typically expected to write notes for patients they care for. As a sensitivity analysis, the number of unique patients with orders placed was assessed as an alternate measure of patient load. The number of ordering sessions was calculated based on the total number of times that the “Order list changed” audit log metric was identified in the clinician’s audit log sequence on a given day.

As the study included data from a 3-month period, each clinician contributed multiple clinician-days to the overall dataset; each clinician-day was recorded as a unique observation in the dataset. As such, the data were clustered at the clinician level (each clinician contributing multiple clinician-days over the study period) and at the clinician service level (in which multiple clinician-days from different clinicians could be from the same clinical service). These levels were not nested because a specific clinician may have had clinician-days in several clinical settings. Finally, RAR events from the 3-month period were organized as a binary variable by clinician-day. The occurrence of more than 1 RAR event on a given day was rare (occurring on <0.01% of clinician-days), and there were never more than 2 RAR events on any included clinician-day. Therefore, examining the presence of any RAR event (even if multiple occurred) on each clinician-day was determined to be the most suitable analytic approach.

### Measures and Outcome

The primary exposure variable was secure messaging volume, calculated as the number of secure messages sent and received by a clinician per day. Clinicians were categorized by their role as attending physician, trainee physician, or APP. Additional covariates included the clinician age in years, clinician sex, daily patient load, total ordering sessions, and clinical service assignment. Data on clinician race and ethnicity were not collected. The primary outcome was a binary indicator of whether a clinician made a wrong-patient ordering error on a given clinician-day (based on the presence of an RAR event).

### Statistical Analysis

Descriptive statistics were calculated as median (IQR) values or as frequencies and percentages. To determine the association between secure messaging volume and wrong-patient ordering events, we used a multilevel logistic regression model. The model included a linear term for secure messaging volume as the primary exposure variable and adjusted for clinician age, sex, clinical role, patient load, and ordering sessions as fixed effects. A random effect for individual clinicians was included to account for correlations among individuals. A random effect for clinical service assignment was used to account for similarities in work patterns within settings. Odds ratios (ORs) and 95% CIs were calculated. An additional model including an interaction term between secure messaging volume and clinician role was assessed. For this model, ORs and 95% CIs were stratified by clinician role using custom hypothesis test statements.

A 2-sided *P* < .05 was considered the threshold for significant. All analyses were performed using R, version 4.3.3 and RStudio, version 2023.12.1 (R Foundation).

## Results

### Sample Characteristics

A total of 75 546 clinician-days from 3239 unique clinicians (median [IQR] age, 37 [32-46] years; 1791 female [55.3%] and 1448 male [44.7%]) performing 124 941 746 audit log actions, with 329 unique clinical service assignments, were included in the study (Table 1). The median number of EHR audit log actions observed per day was 1400 (IQR, 925-2074). The sample consisted of 1680 attending physicians (51.9%), 999 APPs (30.8%), and 560 trainee physicians (17.3%). The median patient load across all clinician-days was 6 (IQR, 3-10) patients per day, the median EHR time was 294.8 (IQR, 194.6-410.4) minutes per day, and the median number of ordering sessions was 17 (IQR, 6-39) per day. The median secure messaging volume was 16 (IQR, 0-61) messages per day, and 24 642 clinician-days (32.6%) had no secure messaging use. Only 295 clinician-days (0.4%) involved the occurrence of 1 or more RAR errors. The distribution of secure messaging volume across clinician-days is shown in eFigure 1 in Supplement 1, and the variability of secure messaging volume over time within clinicians is shown in eFigure 2 in Supplement 1.

**Table 1.** Study Cohort Characteristics, Stratified by Clinician Role (N = 3239)

Characteristic	Median (IQR)		
	Attending physician (n = 1680)	Trainee physician (n = 560)	APP (n = 999)
Age, y	39 (34-49)	30 (28-32)	39 (34-48)
Sex, No. (%)			
Female	684 (40.7)	275 (49.1)	832 (83.3)
Male	996 (59.3)	285 (50.9)	167 (16.7)
Total clinician-days, No.	33 806	17 682	24 058
Total EHR time, min/d	265.5 (167.0-401.2)	307.0 (209.9-412.2)	317.6 (225.9-419.1)
Patient load, patients/d	7 (3-12)	6 (3-8)	6 (3-9)
Ordering sessions, orders/d	15 (5-35)	20 (7-38)	19 (5-43)
Secure messaging volume, messages/d	8 (0-43)	41 (8-96)	15 (0-55)
Clinician-days with no secure messages, No. (%)	13 354 (39.5)	3256 (18.4)	8032 (33.4)
Secure messaging volume among days with any messages, messages/d	31 (11-83)	58 (25-114)	37 (15-79)
Clinician-days with RAR events, No. (%)	109 (0.3)	81 (0.5)	105 (0.4)

Abbreviations: APP, advanced practice practitioner; EHR, electronic health record; RAR, retract and reorder.

### Secure Messaging and Wrong-Patient Ordering Errors

In multivariable analysis, clinician-days with secure messaging at the 75th percentile (61 messages per day) had higher odds of wrong-patient ordering errors compared with those at the 25th percentile (0 messages per day) (OR, 1.10; 95% CI, 1.01-1.20; *P* = .03) (Table 2). Results were not meaningfully changed in sensitivity analyses using an alternate measure (patients with orders changed) of patient load (eTable in Supplement 1). The interaction between secure messaging volume and clinician role was statistically significant (*P* = .03); as such, we generated stratified estimates based on clinician role (Table 3).

**Table 2.** Associations Between Secure Messaging Volume, Patient Load, and Clinician Characteristics and the Presence of Any Wrong-Patient Retract-and-Reorder Event^a^

Variable	Scaled OR (95% CI)^b^	*P* value
Secure messaging volume	1.10 (1.01-1.20)	.03
Patient load	0.92 (0.78-1.08)	.28
Ordering sessions	2.65 (2.33-3.02)	<.001
Clinician role		
Attending physician vs APP	0.84 (0.57-1.26)	.40
Trainee physician vs APP	1.13 (0.71-1.80)	
Clinician sex: male vs female	1.26 (0.89-1.79)	.77
Clinician age	1.04 (0.81-1.33)	.19

Abbreviations: APP, advance practice practitioner; OR, odds ratio.

^a^ Based on a multilevel logistic regression model that controls for repeated measures of individuals over time and clustering within login departments.

^b^ The ORs were scaled to represent a change from the 25th to 75th percentile for secure messaging volume (0-61 messages), patient load (3-10 patients), ordering sessions (6-39 sessions), and age (31-45 years).

**Table 3.** Association Between Secure Chat Volume and the Presence of Any Wrong-Patient Retract-and-Reorder Event, Stratified by Clinician Role^a^

Variable	Scaled OR (95% CI)^b^	*P* value
Secure messaging volume among attending physicians	1.20 (1.02-1.42)	.02
Secure messaging volume among trainee physicians	0.91 (0.74-1.12)	.65
Secure messaging volume among APPs	1.18 (1.00-1.40)	.045
Patient load	0.92 (0.79-1.08)	.33
Ordering sessions	2.65 (2.33-3.01)	<.001
Clinician sex: male vs female	1.25 (0.88-1.77)	.68
Clinician age	1.05 (0.82-1.35)	.21

Abbreviations: APP, advance practice practitioner; OR, odds ratio.

^a^ A multilevel logistic regression model was used, including an interaction term for clinician role and secure messaging volume, adjusting for clinician age, sex, patient load, order volume, and repeated measures within individuals and clustering within login departments.

^b^ The ORs were scaled to represent a change from the 25th to 75th percentile for secure messaging volume (0-61 messages), patient load (3-10 patients), ordering sessions (6-39 sessions), and age (31-45 years).

The association of daily secure messaging volume with wrong-patient ordering errors was strongest among attending physicians (OR, 1.20; 95% CI, 1.02-1.42; *P* = .02), followed by APPs (OR, 1.18; 95% CI, 1.00-1.40; *P* = .045). There was no association between secure messaging volume and errors among trainees (OR, 0.91; 95% CI, 0.74-1.12; *P* = .65). Other covariates, such as clinician age, sex, and patient load, were also not associated with wrong-patient ordering errors (Table 3); however, a greater number of ordering sessions was associated with an increased odds of a wrong-patient ordering error (OR, 2.65; 95% CI, 2.33-3.01; *P* < .001).

## Discussion

We conducted this cohort study to assess the association between daily secure messaging volume and self-intercepted wrong-patient ordering errors. We found that clinician-days with high levels of secure messaging use were associated with higher odds for wrong-patient ordering errors, highlighting the potential implications of increased secure messaging use for patient safety. Although several studies have characterized the use and adoption of secure messaging in health care settings, to our knowledge, this study is the first to assess its association with patient safety.

Reasons for the observed association between daily secure messaging volume and wrong-patient ordering errors are unclear. One possible explanation is secure messaging’s potential contribution to task interruptions, which consequently may contribute to the risk for errors. Although secure messaging is designed to be used asynchronously, the median response time to secure messages has been shown to be short (approximately 2 minutes), suggesting that clinicians may be pausing their ongoing tasks to respond to secure messages.^20,21,22,23^ The association among interruptions, task completion times, and propensity for errors is well established and has been studied in many clinician groups and health care settings.^24,25,26,27,28^ However, it is also possible that high secure messaging volume is merely a reflection of high clinical workload, and such clinical environments are also high-risk environments for errors. We attempted to adjust for clinical workload and environment by adjusting for patient load and clinical service assignments, but the possibility for residual confounding remains.

We found that the strongest association between secure messaging volume and wrong-patient ordering errors occurred among attending physicians, whereas no association was observed among trainee physicians. Notably, attending physicians also had the lowest median daily volume of secure messaging, and trainee physicians had the highest. It is possible that clinicians who use secure messaging more frequently may be less susceptible to its potentially interruptive effects. However, these differences may also be due to different ways that secure messaging may be used by different groups of clinicians. Although trainee physicians were the youngest group in the study (and therefore may be more accustomed to technology use), we did not find a statistically significant association between clinician age and wrong-patient ordering errors.

It is important to highlight that wrong-patient ordering errors (identified through RAR events within the EHR) represent only 1 type of near-miss ordering errors. However, near-miss errors often share the same causal pathways as errors that reach the patient, and the use of near-miss errors has been endorsed by major safety organizations as a way to study and improve health care safety.^29,30,31,32^ Moreover, wrong-patient ordering errors capture a cognitive error^16^ and have been used in investigating the association of distractions and/or cognitive load on errors. For example, wrong-patient ordering errors have been used to assess risks associated with the number of simultaneously open patient charts,^15^ choice of temporary names for newborns,^33^ and multiple vs singleton births in the neonatal intensive care unit^34^ and to compare clinical settings and workload.^35^ In the present study, given the potential role of secure messaging in clinician interruptions (based on several prior studies^20,21,22,23^) and thereby increased attention switching and cognitive burden, studying wrong-patient ordering errors is a promising proxy for assessing patient safety outcomes.

With the exponential growth of secure messaging and its widespread use across health systems, it is imperative to develop guidelines and principles for secure messaging use that decrease clinician burden and maintain patient safety. Toward this end, it is important to consider innovative design interventions that may mitigate the interruptive effects of secure messages. For example, prior research has shown that interruptions may cause the least cognitive burden when they are situated at task boundaries (eg, just after a person has completed a task or subtask).^20,21^ Timing the delivery of secure messages at natural task break points (eg, when closing a patient’s chart or after signing a note or order) using algorithmic or machine learning techniques may mitigate the cognitive load associated with interruptions.

Similarly, research has highlighted that errors arise from interruptions because of the high cognitive effort required in resuming (and reengaging in) the paused original task.^22^ Mechanisms to provide environmental cues (similar to the idea of sticky notes) can provide external memory regarding the state of a paused task, helping clinicians to resume a task with limited cognitive effort.^23^ Such cognitive burden–reducing strategies may mitigate the challenges associated with secure messaging use.

### Limitations

This study has several limitations. First, it was a single-center study and may not be generalizable to other settings. However, we included data from 14 hospitals that represented academic and community settings and included more than 3200 clinicians and more than 75 000 clinician workdays. Second, we could not adjust for patient complexity in this study for several reasons, including a large proportion of the secure messages (approximately 30%) not having associated patient information and a diverse set of inpatient clinical settings making it difficult to develop a uniform measure of patient complexity. Additional analysis with patient characteristics may provide valuable insights on messaging characteristics and various outcomes. Third, the measure for patient load was based on the number of patients for whom the clinician signed a note on that day. Although this measure is an approximation, it is likely correlated with the true patient load and, thus, may provide a meaningful metric for statistical adjustment. We conducted a sensitivity analysis using an alternate definition of patient load and found the results largely unchanged (eTable in Supplement 1). Fourth, clinician login information was used to ascertain the clinical service assignment associated with each clinician-day. Although this measure is a reasonable proxy, recent research has shown that there may be variability among clinician types in the accuracy of this approach.^36^ Fifth, more secure messaging volume is also potentially an indicator of increased coordination and collaboration among clinicians. Thus, the findings from this study, which was designed to assess the association between secure messaging volume and wrong-patient ordering errors at the clinician-day level, cannot provide conclusive evidence regarding the direct impact of messaging on errors. It is possible that some of the messages may have assisted in helping clinicians to retract orders (as measured by RAR events); however, we did not have the content of the messages to assess this possibility. Finally, the exact number of wrong-patient ordering errors that reached the patient in this study is difficult to estimate; however, as previously described, near-miss errors have the same causal pathways, and a recent study estimated that approximately 2% of wrong-patient ordering errors reach the patient.^37^

## Conclusions

In this cohort study of inpatient clinicians, clinician-days with high secure messaging volume were associated with higher odds of wrong-patient orders. Although preliminary, these findings may have implications for patient safety. With secure messaging applications being rapidly adopted across US medical centers, it is important to identify mechanisms to streamline their adoption and use.

## Notes

Supplement 1. **eFigure 1.** Distribution of Secure Messaging Volume **eFigure 2.** Secure Messaging Volume Over Time for a Random Sample of Clinicians Included in the Study **eTable.** Results of Sensitivity Analysis Using Total Number of Patients With Orders Changed to Reflect Patient Load, as Opposed to Total Number of Patients With Notes Signed

Supplement 2.
Data Sharing Statement
