# Supplementary material for: Secure Messaging Use and Wrong-Patient Ordering Errors Among Inpatient Clinicians
Source: JAMA Netw Open. 2024 Dec 4;7(12):e2447797. doi: 10.1001/jamanetworkopen.2024.47797 (PMC11618466; doi:10.1001/jamanetworkopen.2024.47797)
Supplement: Supplement 1. — eFigure 1. Distribution of Secure Messaging Volume eFigure 2. Secure Messaging Volume Over Time for a Random Sample of Clinicians Included in the Study eTable. Results of Sensitivity Analysis Using Total Number of Patients With Orders Changed to Reflect Patient Load, as Opposed to Total Number of Patients With Notes Signed [file jamanetwopen-e2447797-s001.pdf]

Supplemental Online Content

Lou SS, Lew D, Xia L, Baratta L, Eiden E, Kannampallil T. Secure messaging use and wrong-patient ordering errors among inpatient clinicians. *JAMA Netw Open*. 2024;7(12):e2447797. doi:10.1001/jamanetworkopen.2024.47797

eFigure 1. Distribution of Secure Messaging Volume

eFigure 2. Secure Messaging Volume Over Time for a Random Sample of Clinicians Included in the Study

eTable. Results of Sensitivity Analysis Using Total Number of Patients With Orders Changed to Reflect Patient Load, as Opposed to Total Number of Patients With Notes Signed

This supplemental material has been provided by the authors to give readers additional information about their work.

© 2024 Lou SS et al. *JAMA Network Open*.

eFigure 1. Distribution of Secure Messaging Volume. A. All clinician-days. B. Clinician-days with any secure messages. C. Clinician-days with any RARs.

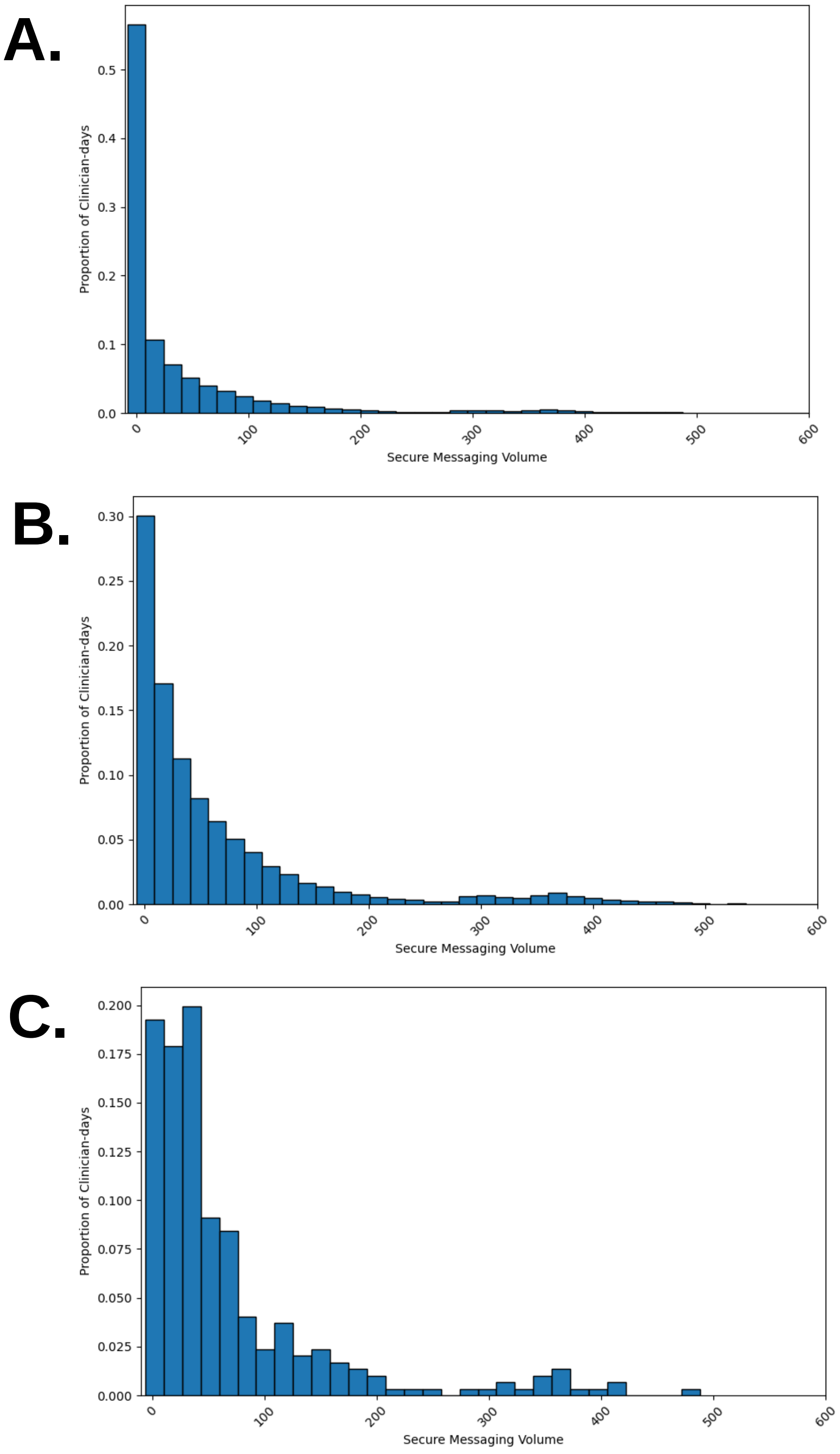

© 2024 Lou SS et al. *JAMA Network Open*.

eFigure 2. Secure Messaging Volume Over Time for a Random Sample of Clinicians Included in the Study

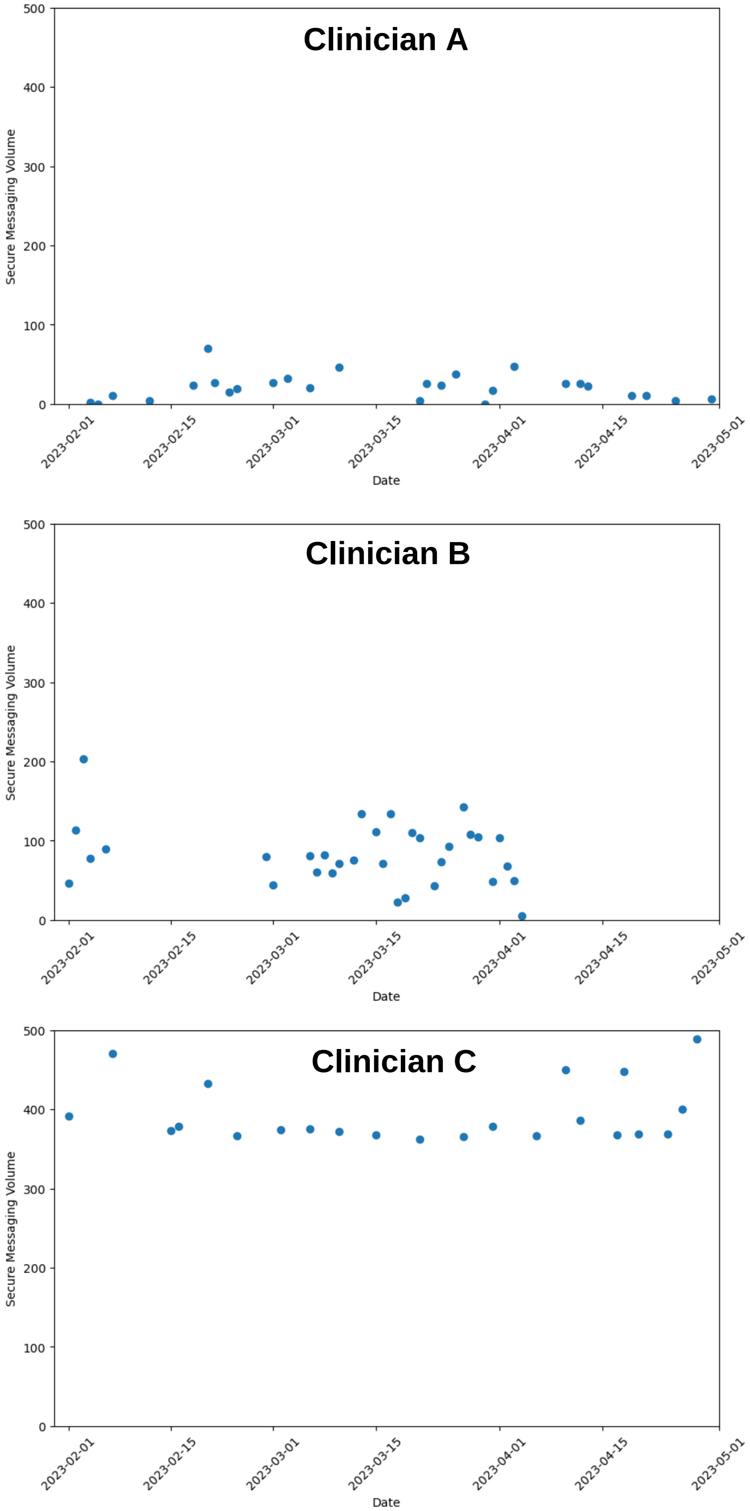

© 2024 Lou SS et al. *JAMA Network Open*.

eTable. Results of Sensitivity Analysis Using Total Number of Patients With Orders Changed to Reflect Patient Load, as Opposed to Total Number of Patients With Notes Signed

A multi-level logistic regression model was used to examine the association between secure messaging volume and any RAR error, adjusting for clinician age, gender, patient load, order volume, and repeated measures within individuals and clustering within login departments; Note: odds ratios were scaled to represent a change from the 25th to 75th percentile for secure messaging volume (0 to 61 messages), patient load (3 to 10 patients), ordering sessions (6 to 39 sessions), and age (31 to 45 years)

**Outcome variable**	**Variable**	**Scaled Effect Estimate (95% CI)***	**p-value**
RAR error	Secure messaging volume	1.09 (1.01, 1.16)	0.020
	Patient load (patients with orders changed)	0.87 (0.72, 1.05)	0.144
	Ordering sessions	2.47 (2.16, 2.83)	< 0.001
	Clinician role: Attending physician vs. APP	0.84 (0.56, 1.25)	0.394
	Clinician role: Trainee physician vs. APP	1.13 (0.70, 1.80)	
	Clinician gender: Male vs. Female	1.26 (0.89, 1.79)	0.192
	Clinician age	1.04 (0.80, 1.34)	0.794

© 2024 Lou SS et al. *JAMA Network Open*.
