# Supplementary material for: Secure Messaging Use and Wrong-Patient Ordering Errors Among Inpatient Clinicians
Source: JAMA Netw Open. 2024 Dec 4;7(12):e2447797. doi: 10.1001/jamanetworkopen.2024.47797 (PMC11618466; doi:10.1001/jamanetworkopen.2024.47797)
Supplement: Supplement 2. — Data Sharing Statement [file jamanetwopen-e2447797-s002.pdf]

Data Sharing Statement

Lou. Secure Messaging Use and Wrong-Patient Ordering Errors Among Inpatient Clinicians. *JAMA Netw Open*. Published December 04, 2024. doi:10.1001/jamanetworkopen.2024.47797

Data
Data available: No
